# Modification of barley dietary fiber through thermal treatments

**DOI:** 10.1002/fsn3.1026

**Published:** 2019-04-09

**Authors:** Huma Bader Ul Ain, Farhan Saeed, Muhammad Asif Khan, Bushra Niaz, Madiha Rohi, Muhammad Adnan Nasir, Tabussam Tufail, Friha Anbreen, Faqir Muhammad Anjum

**Affiliations:** ^1^ Institute of Home & Food Sciences Government College University Faisalabad Faisalabad Punjab Pakistan; ^2^ University of Agriculture Faisalabad Faisalabad Punjab Pakistan; ^3^ Department of Food Science and Technology Government College Women University Faisalabad Faisalabad Punjab Pakistan; ^4^ Department of Allied Health Sciences, Institute of Diet and Nutritional Sciences University of Lahore Gujrat Punjab Pakistan; ^5^ National Institute of Food Science and Technology University of Agriculture Faisalabad Faisalabad Punjab Pakistan; ^6^ The University of Gambia Gambia Gambia

**Keywords:** barley, insoluble dietary fiber, modification, soaking, soluble dietary fiber, thermal treatment

## Abstract

The current research was carried out to observe the effect of different thermal treatments on soluble and insoluble dietary fiber ratio to improve functional properties of barley. Two varieties of barley labeled as Haider‐93 and Jau‐87 were milled and then wet and dry heat‐treated. Soaking and then cooking of soaked and nonsoaked barley was performed. Untreated barley contained more insoluble dietary fiber (12.00–12.40 g/100g dm) than soluble dietary fiber (4.73–5.70 g/100g dm). Additionally, the modification of soluble (13.32%) and insoluble dietary fiber (8.79%) ratio through pressure cooking was nonsignificant while roasting showed significant results, that is, 53.91% increase in soluble dietary fiber and 8.79% decrease in insoluble dietary fiber. In phase II, cooking without soaking gave highest results, that is, 68.08% increase in soluble dietary fiber and 15.48% decrease in insoluble dietary fiber. Conclusively, among all treatments of phase I and II, the better results were shown by cooking without soaking.

## 1 INTRODUCTION

In recent era, dietary fiber is acknowledged as one of the main functional ingredients (Lorencio & Alvarez, 2016; Singh, 2016). In 1953, Hipsley was first to determine the term “dietary fiber” for the nondigestible constituents of plant cell walls (Hipsley, 1953). It is mainly recognized as carbohydrate polymer with ten or more monomeric units, which is resistant to enzymatic digestion in small intestine of humans. It is conventionally classified into two categories according to their water solubility: insoluble dietary fiber and soluble dietary fiber (Borderias, Alonso, & Mateos, 2005; Esposito et al., 2005). The growing interest in dietary fiber is owing to their functional properties and potential health benefits (Jha & Berrocoso, 2015). With this respect, soluble dietary fiber has been found to be significant in reducing the high cholesterol, triglyceride, and glucose levels in blood through binding. These can also affect the texture, gelling, thickening, and emulsifying properties of foods (Anderson, 1986). Whereas, insoluble dietary fiber reduce the transit time of food through intestine owing to their water‐holding capacity. Among foods, peas, beans, fruits, and vegetables are the good sources of soluble dietary fiber, while, grain‐based foods are considered as poor sources. Conclusively, soluble dietary fiber is found to have more functional perspectives than insoluble dietary fiber. But the level of insoluble dietary fiber is more as compared to soluble dietary fiber in cereals and other grain‐based foods. Barley (*Hordeum vulgare* L.) is at fourth position in cereals in worldwide production. It has been a considerable staple food in the Arabian Peninsula since earlier eras, and its applications in the food industry are quite limited. It is used as feed or malt. Although barley is a low cost, food grade cereal fiber source, its use as an ingredient in foods has been relatively unsatisfactory due to its poor functionality. Therefore, the need for the modification of the functional characteristics of cereal grains before their incorporation into foods is evident.

For the purpose, thermal processes are considered as most important approach for the modification of soluble and insoluble fibers ratio and physicochemical properties of dietary fiber (Zhou, Qian, Zhou, & Zhang, 2012). Different methods are used for thermal modification such as sterilization, sun drying, steam processing, boiling, frying mainly deep fat frying, microwave drying, vacuum‐belt drying, roasting, and pressure cooking. These treatments significantly change the content and accessibility of nutrients and ameliorate the physiological effects of these nutrients by changing the plant cell wall composition. The amount of soluble dietary fiber produced is highly dependent on the temperature of the processes. This high temperature breaks the glycosidic bonds in polysaccharide, which can lead to the release of oligosaccharides and thus increase the quantity of soluble dietary fiber (Wang et al., 2015; Yi, Wang, Zhuang, Pan, & Huang, 2014). The effect of steam processing and sun drying on *Polygonatum odoratum* was probed, and it was found that the steam processing significantly increased the oil holding capacity whereas, sun drying significantly increased the water‐holding capacity and swelling power of *P. odoratum* fiber (Lan, Chen, Chen, & Tian, 2012). Moreover, in the study of Yan and Kerr (2013), continuous vacuum‐belt drying (VBD) was applied to the apple pomace at three different temperatures (80ºC, 95ºC, 110ºC).

Keeping in mind all the aforementioned perspectives, there is a need to partially convert this insoluble dietary fiber into soluble dietary fiber and to develop the soluble dietary fiber enriched value added barley products. The objective of current study was to evaluate the comparative effect of thermal treatments on the modification of insoluble dietary fiber into soluble dietary fiber in two barley varieties. The current paper describes the effect of thermal treatments on dietary fiber modification (means conversion of Insoluble dietary fiber into soluble dietary fiber) as soluble fiber has much importance than insoluble fiber. Soluble fiber has a positive role in product development as well as their health benefits. Therefore, after modification, it is much important for an industrial point of view.

## 2 MATERIALS AND METHODS

### 2.1 Procurement of raw material

Two barley varieties, that is Haider‐93 and Jau‐87 were procured from Ayub Agriculture Research Institute (AARI) Faisalabad. Seeds were cleaned to remove any debris or field dirt and sealed in polyethylene bags.

### 2.2 Determination of dietary fiber

The contents of soluble, insoluble, and total dietary fibers in both barley varieties were determined according to the method of AOAC 991.43 enzymatic gravimetric method (AOAC, 2005).

### 2.3 Phase 1

#### 2.3.1 Milling

The cleaned grains were pulverized using a plate mill to obtain whole flour (WF). A part of the whole flour was further sieved through a 44 mesh sieve (BSS). The “+”fraction was termed as the bran rich fraction (BRF), and the “–” fraction was termed as semi‐refined flour (SRF) (Pushparaj & Urooj, 2011).

#### 2.3.2 Wet and Dry Heat Treatment

Each batch of the two commercially available barley varieties was pressure cooked for 10 min (9.8 × 104 Pa) and boiled for 30 min, respectively. The processed grains were dried in an oven at 50˚C and milled into flour. Each of the barley varieties was **roasted** in an open pan for 10 – 15 min at 200˚C and milled into flour (Pushparaj & Urooj, 2011).

### 2.4 Phase 2

#### 2.4.1 Soaking

Soaked barley refers to barley soaked in tap water overnight at room temperature (300 ml of tap water were added to 100 g of barley grains; soaking took place for 18 hr at 20˚C); the soaked grains were drained, dried on a paper towel, lyophilized, ground for 3 min in a coffee mill and stored in polyethylene bags at room temperature prior to analyses (Kutos, Golob, Kac, & Plestenjak, 2003).

#### 2.4.2 Cooked‐soaked barley

Cooked‐soaked barley refers to barley soaked in tap water overnight at room temperature (300 ml of tap water were added to 100 g of barley grains, soaking took place 18 hr at 20˚C) then drained and dried on a paper towel and consequently cooked in fresh tap water (volume ratio beans to water being 1–6) boiling in a covered pot (98–100˚C) until these became suitable for consumption (approx. 40 min). Cooked grains were drained and treated in the same way as soaked barley (Kutos et al., 2003).

#### 2.4.3 Cooked nonsoaked barley

Cooked nonsoaked barley refers to nonsoaked barley directly cooked in boiling water until these became suitable to consumption. Nonsoaked barley (50 g) was cooked in 400 ml of tap water boiling in a covered pot (98–100˚C) until these became suitable for consumption (approx. 2 hr). Cooked grains were drained and treated in the same way as soaked barley (Kutos et al., 2003).

#### 2.4.4 Canned barley

Canned barley (CnB) refers to barley directly from a commercial can, which have been drained and treated in the same way as soaked baryley (Kutos et al., 2003).

### 2.5 Statistical analysis

The data obtained for each parameter were subjected for Latin square design (LSD) to determine the level of significance (Steel, Torrie, & Dickey, 1997).

## 3 RESULTS AND DISCUSSION

### 3.1 Dietary fiber content of barley

The soluble and insoluble fiber contents of the native and all the thermally modified barley of two different varieties were measured. Mean values for dietary fiber content of two barley varieties before treatment were exhibited in Table 1. Results showed that soluble dietary fiber content was higher in Haider‐93 (5.70 g/100 g dm) than in Jau‐87 (4.73 g/100g dm) whereas insoluble dietary fiber was more in Jau‐87 (12.00 g/100g dm) than in Haider‐93 (12.40g/100g dm). Literature showed similar results as of present study. Beloshapka, Buff, Fahey, and Swanson (2016) explicated that barley contained about 8.6%, 4.8%, and 13.4% insoluble, soluble, and total dietary fiber, respectively.

**Table 1:** Mean values for dietary fiber content of thermally treated barley varieties

Treatments	Jau−87			Haider−93		
	SDF	IDF	TDF	SDF	IDF	TDF
Control	4.73^g^	12.40^a^	17.13^c^	5.70^g^	12.00^a^	17.70^e^
Boiling	7.15^c^	9.37^g^	16.52^d^	8.65^h^	9.07^h^	17.72^e^
Pressure cooking	5.36^b^	11.31^b^	18.59^a^	6.12^b^	10.82^b^	16.94^a^
Roasting	7.28^f^	11.31^b^	16.67^d^	8.73^f^	11.00^c^	19.73^g^
Soaking	6.67^d^	9.48^f^	16.15^e^	7.92^c^	9.15^g^	17.07^f^
Cooked‐Soaked barley	6.39^d^	10.91^c^	17.3^b^	7.61^e^	10.68^d^	18.29^c^
Cooked nonsoaked barley	7.95^a^	10.48^e^	18.43^a^	9.05^a^	10.07^f^	19.12^b^
Canned	6.39^e^	10.80^d^	17.13^c^	7.69^d^	10.38^e^	18.07^d^

Note

Means carrying same letter are significantly identical.

Table 1 exhibited the mean values of dietary fiber content in thermally modified varieties of barley. Barley varieties were thermally treated in two phases. In 1st phase, barley varieties were milled and then wet and dry heat‐treated through many ways including boiling, pressure cooking, and roasting. Results of pressure cooking revealed that it slightly modified the soluble and insoluble dietary fiber ratio, that is, soluble dietary fiber was slightly increased and insoluble dietary fiber was slightly decreased in both barley varieties. In Jau‐87, soluble dietary fiber was increased from 4.73 to 5.36 g/100 g and insoluble dietary fiber decreased from 12.40 to 11.31 g/100 g, whereas, in Haider‐93, increase in soluble dietary fiber was from 5.70 to 6.12 g/100g, and decrease in insoluble dietary fiber was from 12.00 to 10.82 g/100 g. This modification was not significant (*p* > 0.05).

**Figure 1:**
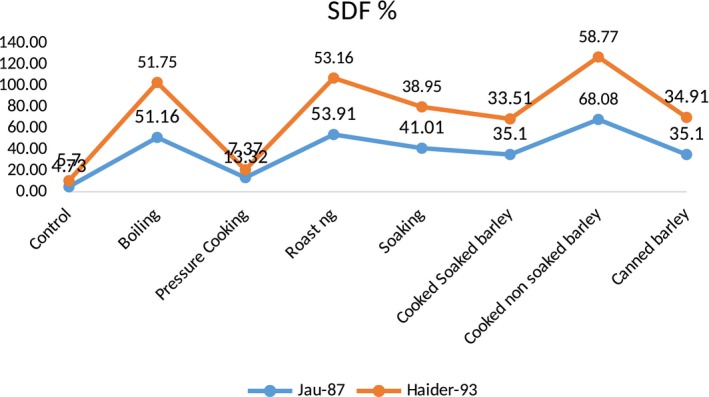
SDF content of thermally modified barley

**Figure 2:**
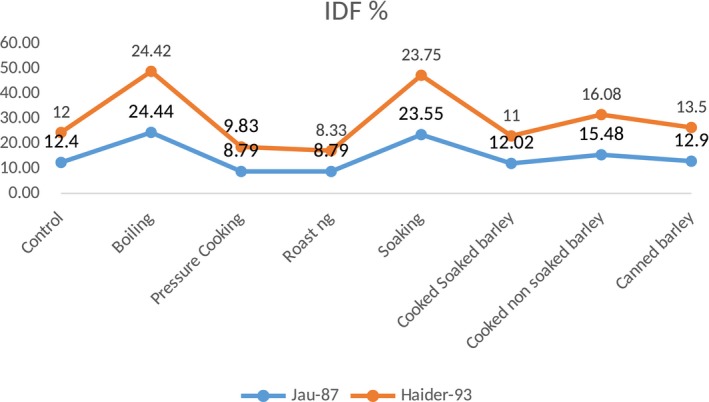
IDF content of thermally modified barley

Moreover, when both varieties were boiled and roasted, these significantly modified the soluble and insoluble dietary fiber ratio. The results of boiling were 51.16% increase in soluble and 24.44% decrease in insoluble dietary fiber in Jau‐87 while, in Haider‐93, 53.91% increase in soluble and 8.79% decrease in insoluble dietary fiber, respectively. When the dietary fiber was roasted, it gave much better results, that is, in Jau‐87, 53.91% increase in soluble and 8.79% decrease in soluble dietary fiber, whereas 53.16% increase in soluble and 8.33% decrease in insoluble dietary fiber as shown in Figures 1 and 2. It was concluded after 1st phase that roasting significantly increased the soluble dietary fiber while boiling was on top in significantly decreasing the insoluble dietary fiber content among all wet and dry heat treatments.

In 2nd phase, barley varieties were firstly soaked and then cooking of soaked and nonsoaked barley was performed. Canning of both barley varieties was also included in this phase. The results of soaking revealed significant modification of soluble and insoluble dietary fiber ratio. In Jau‐87, soluble dietary fiber was increased from 4.73 to 6.67 g/100 g (41.01%) and insoluble dietary fiber decreased from 12.40 to 9.48 g/100 g (23.55%), whereas, in Haider‐93, increase in soluble dietary fiber was from 5.70 to 7.92 g/100 g (38.95%) and decrease in insoluble dietary fiber was from 12.00 to 9.15 g/100 g (23.75%).

Furthermore, when the soaked barley was cooked, the results were less significant than soaking, that is, 33.51%–35.10% increase in soluble and 10.91%–11.00% decrease in insoluble dietary fiber in both barley varieties. These results were almost similar to the findings of canned barley, that is, 34.91%–35.10% increase in soluble dietary fiber and 12.90%–13.50% decrease in insoluble dietary fiber. Moreover, cooking of nonsoaked barley more significantly modified the soluble and insoluble dietary fiber ratio, that is, soluble dietary fiber was significantly increased and insoluble dietary fiber was significantly increased. In Jau‐87, soluble dietary fiber was increased from 4.73 to 7.95 g/100 g and insoluble dietary fiber decreased from 12.40 to 10.48 g/100 g, whereas, in Haider‐93, increase in soluble dietary fiber was from 5.70 to 9.05 g/100 g, and decrease in insoluble dietary fiber was from 12.00 to 10.07 g/100 g. This modification was significant (*p* < 0.05).

Among all treatments, the highest results were shown by cooking without soaking. Therefore, it was concluded that thermal processes can also change the ratio of soluble and insoluble fibers and physicochemical properties of dietary fiber (Zhou et al., 2012). These modify the composition and availability of nutrients. These also modify the plant cell wall material that may have important physiological effects. The amount of soluble dietary fiber produced is highly dependent on the temperature of the processes. This high temperature breaks the glycosidic bonds of polysaccharide which can lead to the release of oligosaccharides and thus increase the quantity of soluble dietary fiber (Wang et al., 2015; Yi et al., 2014).

## 4 CONCLUSION

Both varieties of barley were found to be very high‐quality reservoir of insoluble dietary fiber and poor resource of soluble dietary fiber. Through thermal treatments application, barley was modified with respect to soluble dietary fiber, insoluble dietary fiber ratio, that is, soluble dietary fiber was increased, and insoluble dietary fiber was decreased. Although all treatments had given effective results, cooking without soaking was most effective. This modification opens the door for the betterment of physiochemical, physiological, and functional properties of dietary fiber by increasing the soluble dietary fiber. As a promising source of soluble dietary fiber, dietary fiber should be exploited for therapeutic and health‐enhancing food products.

## CONFLICT OF INTEREST

Authors declare that they have no conflict of interest.

## ETHICAL STATEMENT

This article does not contain any studies with human participants or animals performed by any of the authors. It is further certified that human and animal testing is unnecessary in this study.

## INFORMED CONSENT

For this type of study, formal consent is not required.
